# Nanosatellite Payload for Research on Seed Germination in a 3D Printed Micropot

**DOI:** 10.3390/s23041974

**Published:** 2023-02-10

**Authors:** Bartosz Kawa, Patrycja Śniadek, Rafał Walczak, Jan Dziuban

**Affiliations:** Department of Microsystems, Faculty of Electronics, Photonics and Microsystems, Wroclaw University of Science and Technology, 50370 Wroclaw, Poland

**Keywords:** 3D-printed sensor space application, space autonomous laboratory, additive manufacturing, nanosatellite, payload

## Abstract

In this paper, an autonomous payload proposal for a nanosatellite mission allowing for the cultivation of grains in space was presented. For the first time, a micropot made with 3D printing technology, enabling the parametric determination of plant growth, both on Earth and in the simulated microgravity condition, was presented. A completed system for dosing the nutrient solution and observing the growth of a single grain, where the whole size did not exceed 70 × 50 × 40 mm^3^, was shown. The cultivation of *Lepidium sativum* seeds was carried out in the developed system, in terrestrial conditions and simulated microgravity conditions, using the RPM (Random Position Machine) device. The differences in plant growth depending on the environment were observed. It could be seen that the grains grown in simulated microgravity took longer to reach the full development stage of the plant. At the same time, fewer grains reached this stage and only remained at the earlier stages of growth. The conducted research allowed for the presentation of the payload concept for a 3U CubeSat satellite for research into the development of plants in space.

## 1. Introduction

In the beginning, nanosatellite missions were focused on educational aspects [1]. In recent decades, the technology matured, and today the number of nanosatellites developed and launched has been increasing exponentially [1,2]. Some of them are still educational projects, but most are research missions enabling a wide variety of research works, including biology and astrobiology, astronomy, atmospheric science, pharmaceutical research, Earth observation, telecommunication, and material science [1,2]. Nanosatellites are a low cost and a relatively easily available alternative for microgravity and deep space experiments, in comparison to traditional satellite missions or International Space Station (ISS) facilities. Due to the planned potential establishment of bases on the Moon or Mars, the effects of microgravity and deep space environments on biology need to be examined [3].

A comprehensive review on nanosatellite (also often called CubeSat) missions for microbiology and astrobiology research was provided by L. Zea et al. [4]. To date, NASA’s Ames Research Center was the only institution to have operated nanosatellites with live biological payloads. These missions are GeneSat-1 (2006), PharmaSat (2009), O/OREOS (2010) [5], SporeSat-1 (2014), EcAMSat (2017), and recently, BioSentinel (2021) [6,7]. The goals of the missions were to measure *Escherichia coli* protein expression and microbe tracking, the dose–response of *Saccharomyces cerevisiae* fungus, the six-month survival of *Bacillus subtilis*, the gravitational response of *Ceratopteris richardii*, antibiotic resistance on microgravity versus dose for uropathogenic *E. coli*, and to investigate the DNA damage response to deep-space radiation in a eukaryotic organism. The common features of all these missions are experiments on small microorganisms combined with appropriate temperature control, miniaturised optical detection instrumentation, autonomous experiment activation and termination, and data acquisition and transmission to Earth. Most importantly, the key issue that led to the success of the listed missions was the application of microfluidic cards, also known as lab-on-a-chip platforms. None of the nanosatellite missions reported in the literature involved research works on plant growth or even seed germination under microgravity. 

### Growing Plants in Space

Space plant growth experiments have a history of over 40 years [8]. The review paper by P. Zabel et al. describes the different designs and technological solutions implemented in higher plant growth under simulated (on the ground) and true (space) microgravity [8]. On-the-ground, simulated microgravity environments can be obtained by various techniques: changing or randomising the gravity vector (gravitropic stimulus), or by using centrifuges (hyper-gravity), clinostats, or random positioning machines (RPMs). The most realistic “on Earth” microgravity can be obtained by plane free fall [9]. There are numerous studies that have investigated the comparability between ground-simulated microgravity (or rather weightless) platforms with true microgravity experiments [10,11]. The experiments to date suggest that, while these ground devices may be useful tools in some cases, there are some differences observed between plants that grow and develop on these devices and plants that are grown in weightlessness during spaceflight [10,11]. However, the RPM can be a useful proxy of weightlessness for certain biological parameters, as shown in studies with plant cells [10,11]. In addition, due to the difficulty, availability, and cost of spaceflight experiments, the RPM may, in fact, be one of the best substitutes, especially when this instrument can potentially generate results comparable to those from true microgravity [10]. On the other hand, numerous experiments on microgravity plant growth were carried out for decades in space during short-duration crew missions (e.g., Shuttle, Shenzou), as well as those typical for a longer duration onboard space stations (e.g., Salyut, Mir, SkyLab or ISS). The size and shape of plant growth chambers have changed over the last few decades. However, those applied on the ISS are in the form of bulky cassettes compatible with mid-deck lockers which could be installed into, for example, an EXPRESS Rack. The ISS is now the only, most expensive, and most long-term low-gravity platform to perform plant growth experiments. Large satellites offer an excellent quality of microgravity that is not affected by docking maneuvers or crew movement, but are still expensive and with limited availability. Thus, nanosatellite experiments enabling plant growth seem to be attractive to the missions mentioned above. Combined with preliminary ground research works utilising various techniques of microgravity simulation (e.g., RPM), with a reference growth of under 1 g gravity, they enable comprehensive and low-cost research works on microgravity influence on the growth of various plants.

In this paper, we present an autonomous nanosatellite payload based on a 3D-printed micropot. As space experiments are expensive and limited access to space infrastructure is provided, nanosatellite-based astrobiological scientific experiments are alternatives. The first issue in these experiments is a development of the nanosatellite payload, where limited volume, limited power consumption, space radiation, temperature and illumination control, and microgravity are preliminary challenges. Some of these technical challenges are addressed in this paper. The first result describing a 3D-printed micropot was described by us in [12]. Presented in the previous work are positive results and a characterization of the micropot as a novel research tool, which are continued in this work towards a complete system able to autonomously perform a scientific experiment under microgravity conditions. As a result, we developed a research instrument—technically and preliminarily biologically verified—for further biological experiments by research groups specialising in plant growth in microgravity. 

## 2. Materials and Methods

### 2.1. The Nanosatellite Payload

**Figure 1:**
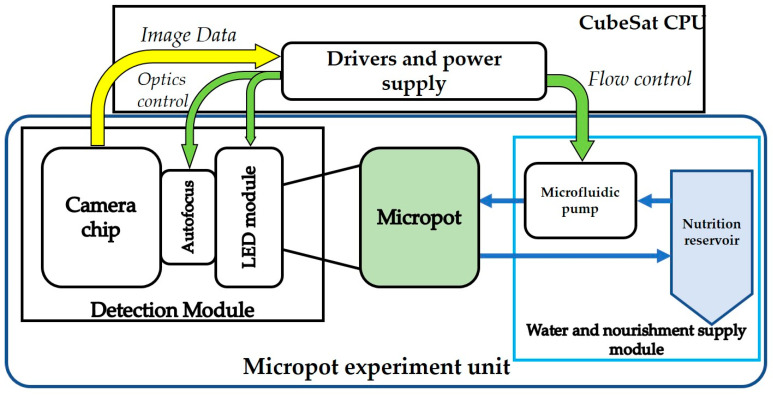
Scheme of the micropot nanosatellite payload.

The nanosatellite payload was designed to fully, automatically, and autonomously sustain and monitor seed growth. The system consists of four main modules: a 3D-printed microsystem device called a micropot, capable of seed growing and biological potential determination; a nutrition supply system (in this experiment, we used water); an optical detection and illumination module; and finally, drivers and a power supply module (Figure 1).

### 2.2. Micropot Construction and Fabrication

**Figure 2:**
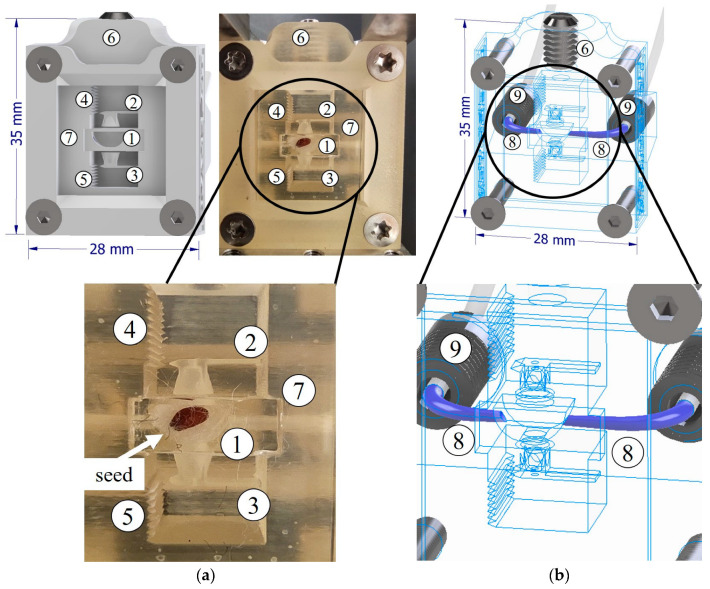
The 3D-printed micropot for a nanosatellite payload: (**a**) CAD design and assembled micropot prepared for the experiment with a seed placed in the socket; and (**b**) Transparent model of the micropot that shows the position of the microfluidic channels. (1) Seed socket; (2) stalk force sensor; (3) root force sensor; (4) force sensor deflection scale; (5) root sensor deflection scale; (6) semi-permeable air supply; (7) protecting glass window; (8) integrated 3D-printed microfluidic channel; and (9) microfluidic connector.

The main component of the payload is the 3D-printed micropot. The overall micropot concept was recently developed by Kawa et al. as a novel tool for the real-time monitoring of seed growth and biological potential assessment experiments with integrated force sensors [12]. Our previous work showed the possibility of performing biological experiments with a semi-automatic system and under 1 g gravity. In the case of microgravity experiments, the micropot and the fluid-based nutrition circuit had to be redesigned to meet all the above conditions of a closed-loop fluid flow in microgravity, and gas exchange in a closed payload container. The printed micropot contains four main components: a seed sample socket, a microfluidic nutrition supply system, a semi-permeable air supply membrane, and two calibrated force sensors (Figure 2).

The micropot was fabricated in a single printing process with a ProJet3510 inkjet printer (3D Systems Inc., Rock Hill, South Carolina, United States) configured to high-definition printing mode. The resolution was 650 × 650 dpi with a 16 µm layer thickness. VisiJet M3 crystal was used as a building material and the wax-like VisiJet S300 as a support material. Postprocessing included four steps. First, the support material was melted away in the oven (60 °C, 2.5 h). Second, the structures were cleaned with mineral oil at 60 °C with ultrasonic agitation (~15 min). Third, the micropots were washed with deionised water (15 MΩcm) and dried in a stream of nitrogen (N_2_). The final step was rising with isopropyl alcohol and drying, which was performed immediately before placing the seeds in the sockets.

The 3D-printed device was equipped with calibrated force sensors [12]. When the root and stalk develop properly, they come into contact with the force sensors. The seed socket has an aperture that, under 1 g gravity conditions, guide the root towards the force sensor. The sensors were calibrated with deflection-force characteristics. Thus, the measurement of the sensor beam deflection can be used to determine the force generated by the root/stalk, and as a result, the biological potential of the seed can be determined. The deflection scale is integrated into the micropot structure, and the displacement is measured with dedicated image analysis software, which automatically tracks the tip of the beam [12]. The micropot was designed to hold a specific kind of seed, which was cress seeds (*Lepidium sativum*). Nevertheless, the 3D printing fabrication process enables the free modification of the seed socket size and the performing of experiments on most small grains.

In order to ensure the gas exchange between the inside volume of the micropot and the tight payload container, the micropot has a semi-permeable air supply membrane made of Parafilm^®^ M, and is located on the top of the micropot. Additionally, the whole device was designed and successfully tested as a robust system able to sustain vibration and overloads of launching into Earth’s orbit.

### 2.3. Monitoring and Nourishment Modules

#### 2.3.1. Optical Monitoring Module

**Figure 3:**
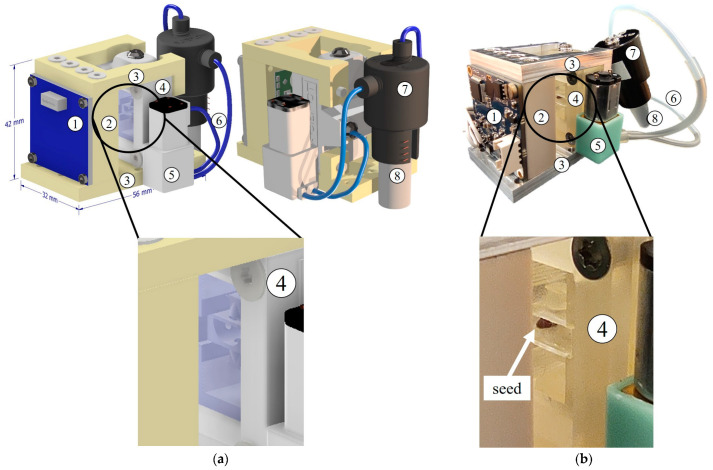
Assembled micropot payload with optical and nutrition modules: (**a**) CAD visualisation; and (**b**) ready to use set-up. (1) Camera chip, (2) LED and autofocus housing, (3) micropot detection module frame, (4) 3D-printed micropot, (5) microfluidic peristaltic pump, (6) microfluidic tubes, (7) Eppendorf fluidic connector, and (8) 1.5 mL Eppendorf enclosure.

The main criteria when designing the monitoring module were the application of a high-resolution image sensor, a large area of observation, and small dimensions (42 mm width, 32 mm length and maximum 15 mm thickness), due to the limited overall available volume in the payload. The detection module was based on an inverted-lens microscope and consisted of: a CMOS camera chip, an autofocus actuator, and an LED illumination circuit. The resolution of the camera chip (Sony IMX179, Japan) was 3264 × 2448 pixels (8 MP). Due to the high magnification and short depth of field, there was a small distortion on the outside perimeter of the picture, but it did not affect the seed observation area quality. Electrically driven autofocus (Logitech C615 Portable HD Webcam) view depth adjustment was in the range of 0.1 mm to 2 mm. The micropot was illuminated with 4 white LEDs, assembled on a dedicated PCB around the autofocus unit. The white light ensured proper irradiation during the seed germination and growth, and illumination during the image capture of the micropot. The total optical power of the LEDs was 0.5 mW. The assembled optical monitoring module thickness was equal to 14 mm, and it was integrated with the micropot module, as presented in Figure 3.

#### 2.3.2. Nourishment Module

One of the most important issues in a controlled seed growth is to obtain precise and time-controlled dosing of the liquid nutrient. The seed requires a moist environment to grow. On the other hand, too much water causes the grain to rot. Additionally, long time gaps in the water supply will cause the grain to dry out and inhibit growth. 

The nourishment module was a closed-loop fluidic hermetic circuit. It contained a microfluidic 1.5 mL Eppendorf reservoir (Elveflow, France), a microfluidic peristaltic micropump (Takasago, Japan), and microfluidic tubing with an internal diameter of 0.5 mm. The total volume of the liquid was less than 2 mL. The water was pumped through the micropot by the micropump and sucked back to the reservoir by pressure generated in the tank. All the microfluidic seals were realised with dedicated microfluidic tube glue to ensure leakage-free connections. The pump was voltage-driven and steered by a microcomputer that emulated the CubeSat control unit (CPU).

### 2.4. Microgravity Simulation 

The RPM (Random Position Machine) was designed and fabricated to sustain two units (2 U–10 × 10 × 20 cm^3^) of experiment payload. The device is capable of different rotation speed adjustments (1–20 rpm). In the case of microgravity simulation, it is crucial to change the direction of the gravity vector over a specific period of time. Various information regarding the rotational speed settings, depending on the conducted experiments, can be found in the literature [13]. For the cultivation of various types of grains, the most commonly used values are between 2 and 10 rpm [14]. In our experiment, we decided to take the value of 5 rpm as the middle value from the range.

**Figure 4:**
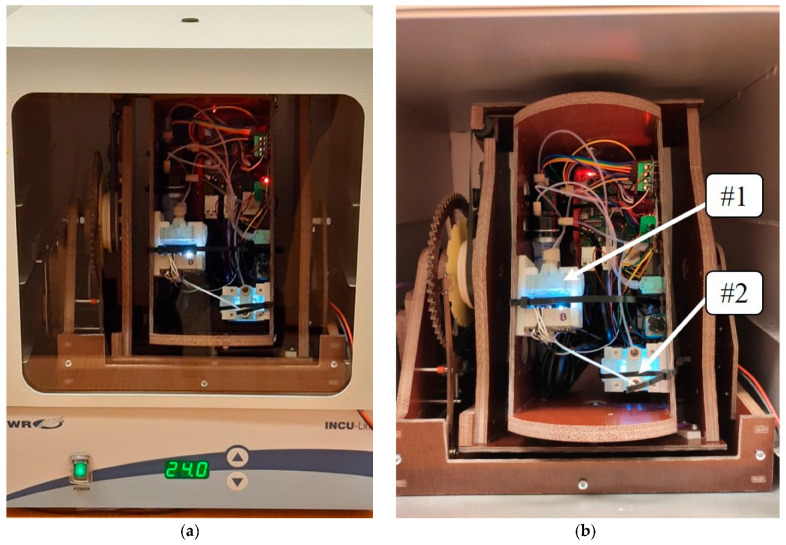
RPM machine with payload: (**a**) during microgravity experiments inside the incubator, and (**b**) two independent micropot modules highlighted on the right picture.

The overall dimension of the RPM with the nanosatellite payload was adjusted to fit a 20 l laboratory incubator (VWR INCU-Line IL23, Belgium) with a stabilised temperature inside (24 ± 1 °C) (Figure 4) and steering/power supply connections. 

Additionally, the payload construction has undergone vibration tests on the vibration exciter (LDS V406, Bruel&Kjear, Denmark) with various frequencies, in a range of 10 to 200 Hz (most common frequencies during the rocket launch), with a magnitude of 10 g. No damages to the payload were noticed. After the vibration tests, the payload was fully functional.

## 3. Results and Discussion

### Microgravity and Reference Experiments 

The microgravity influence on the seed, and further on plants itself, is not well understood yet. Plants have complex regulation mechanisms that stop seed germination in unfavourable environment. Almost all plants exhibit very high gravitropism. The seed root is equipped with specialised cells called statocytes, which detect the vector of gravity at the early stage of germination. Under the microgravity conditions, it was shown [15] that the seeds reveal the so-called automorphogenesis—the process of spontaneous curvatures of roots, followed by straight root elongations in random directions [15]. Other works also exhibited the tendency of the seed root tip to grow in a disoriented manner, with numerous bends, coils, and skews under microgravity conditions. These phenomena are not well understood and require a lot of experimentation for in-depth understanding of the influence of the microgravity on the seed and plant growth [16]. 

In this work, *Lepidium sativum* (garden cress) was selected as the model grain used in many studies, e.g., the influence of microplastics on crops [17]. The best quality grains were selected, i.e., with undamaged husks, oval-shaped, flattened, and characterised by a reddish-brown colour. Each of the seeds were placed in the socket of the disposable 3D-printed micropot to allow for growth. There was a substrate (cotton wool) for cultivation in the socket to ensure the maintenance of moisture during the growth process. The fluidic nutrient (water) in the microfluidic system was pulsed in two phases. 

Phase “one” was to pump the liquid for 25 s with the set of PWM coefficients for the pump at the level of 30%, which ensured a flow of 500 µL/min. The second phase was no pumping, and it lasted 12 h.

This two-phase nutrition allowed the adequate moisture for proper germination while not overflowing the seed. The micropot was illuminated by white LEDs during the experiment all the time (24 h/7 d).

In total, 32 seeds were investigated: 16 cress seeds in the RPM device (simulated microgravity) and 16 reference seeds (1 g gravity). The cultivation time of each seed lasted 12 days (288 h) and was identical for the tests in the RPM device and the reference conditions. During the growth process, photos of the micropot were taken every 30 min and stored in the CPU. 

The analysis carried out was based on the collected photos and allowed for the identification of five stages of seed growth. A zero stage (“0”) indicated the start of the experiment, when the seed was dry. During this stage, the first portion of the nutrient was delivered. The first stage (“1”) was germination. The essence of this stage is to prepare the seed for growth. This stage is characterised by the grain swelling. The second stage (“2”) is the crack of the husk and the beginning of the root growth. In the third stage (“3”) a leaf appeared. A stage “W” (wilt) was also highlighted. In the “W” stage, the seeds wilted, despite nutrient supply and illumination.

**Figure 5:**
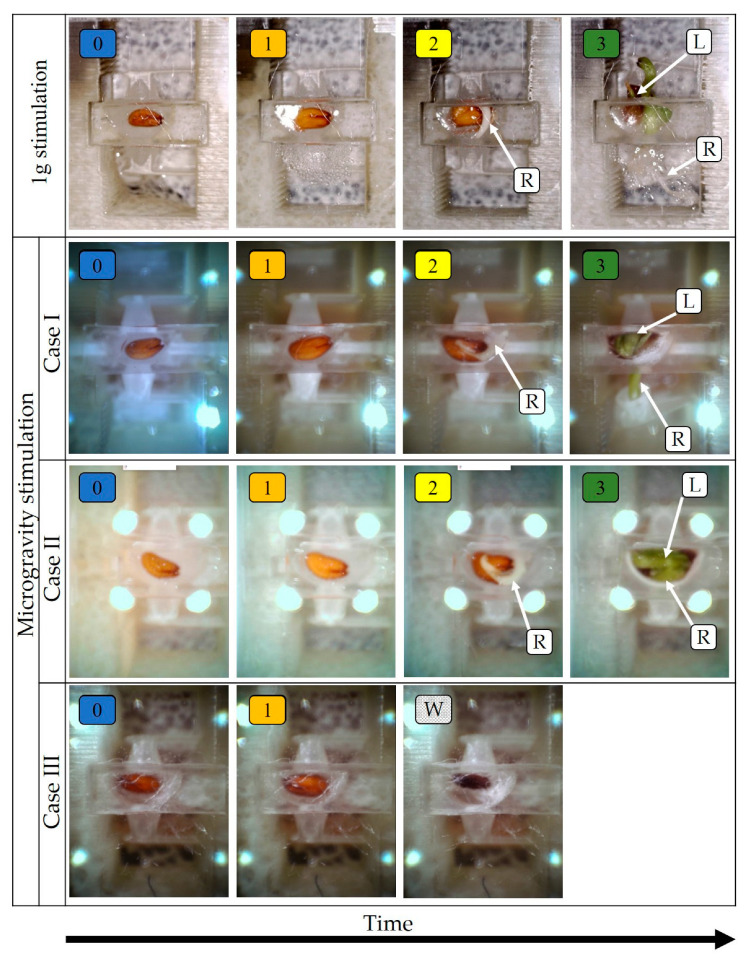
Seed growth stages during the experiment for exemplary grains: (0) beginning of the experiment (initial dry seed, start of water delivery); (1) grain swelling; (2) the appearance of the root; (3) the appearance of the leaf; and (W) seed wilt. The root (R) and leaf (L) are clearly visible at the end of the germination process.

On the base of the captured micropot images (Figure 5), it was possible to notice differences in the stages of growth, depending on whether the seed was grown in the RPM (simulated microgravity, three noticed cases) or reference conditions (1 g gravity). For the experiments carried out at 1 g, each of the distinguished stages lasted much shorter than those for the experiments carried out with the RPM (Figure 5). A significant difference is also the lack of the occurrence of the “W” stage for the reference experiments.

A total of three experiment routes (cases) were identified for the microgravity tests. In case I, all stages of seed growth were achieved, and both root and stalk growth were noticed. The root reached the microbeam force sensors through a hole in the seed socket. In case II, the root was formed, but it did not go through the hole. The root curled in the seed socket. In case III, after the initial germination, the seed wilted.

**Figure 6:**
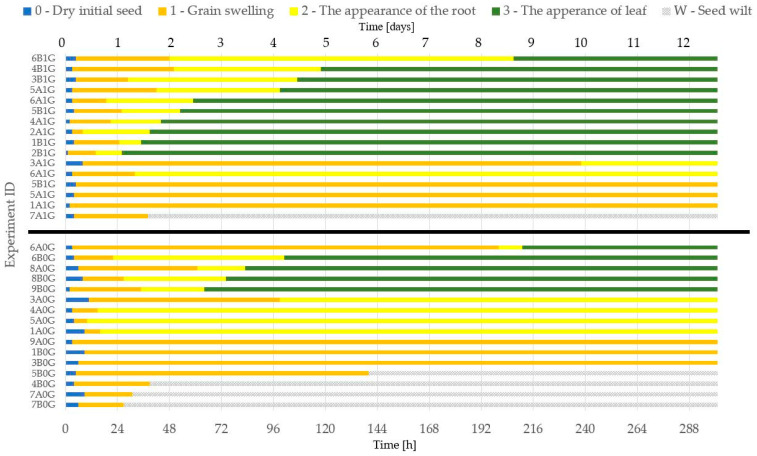
Comparison of seed growth stages in time from the experiments, conducted in 1 g gravity (upper part) and microgravity (lower part) stimulations.

**Figure 7:**
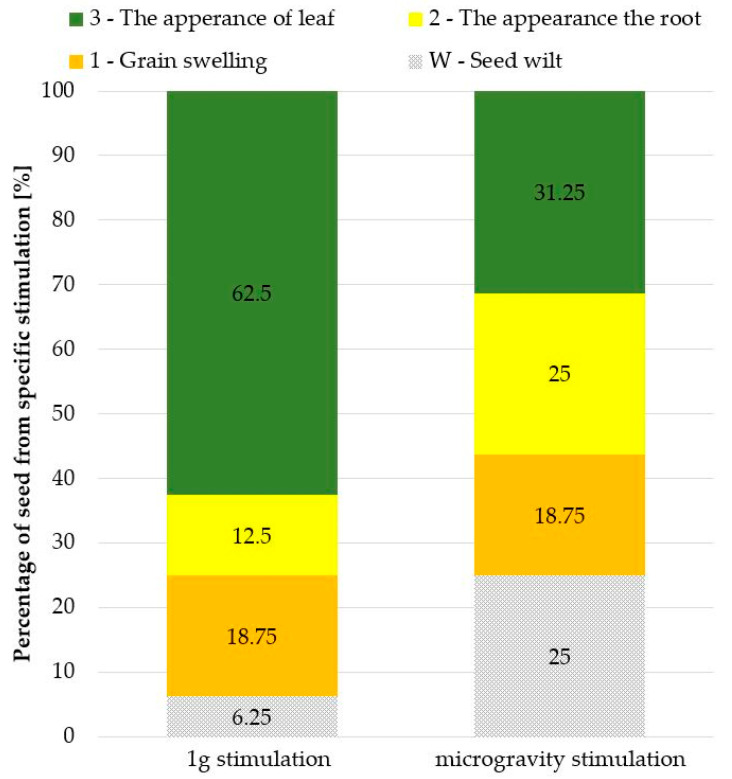
Percentage comparison of growth phases achieved under 1 g stimulation or microgravity.

A comparison of all experiment results as a function of growth time, with the identified stages of the growth, is shown in Figure 6. In the reference experiments, a much larger percentage (more than 60%) of the seeds germinated and passed to the last stage of development and formation of a correct plant (Figure 7).

On the other hand, under the microgravity stimulation, a significant amount of seeds finished their growth in stage “2” or “W”, and only 30% reached the stage of full growth. It was also observed that in the RPM experiments, the leaf and the root were, in most cases, twisted inside the micropot socket, while 1 g stimulation allowed these parts of the plant to grow vertically (according to Earth’s gravity direction). It can be seen that the conditions of simulated microgravity disrupted the proper growth of plants, significantly influencing the endogenous factors (phytohormones and growth regulators), most likely by disturbing the functioning of the specialised cells called statocytes [15], as mentioned at the beginning of this section.

The obtained results are in good agreement with other plant microgravity growth experiments carried out both in simulated and real microgravity conditions through the use of non-miniaturised instrumentations [18], and show that the described experimental platform enables the testing of seeds in simulated microgravity or space conditions. Nevertheless, further biological experiments by research groups specialising in plant growth in microgravity are necessary for an in-depth analysis of the behaviour of the plant in described conditions.

**Figure 8:**
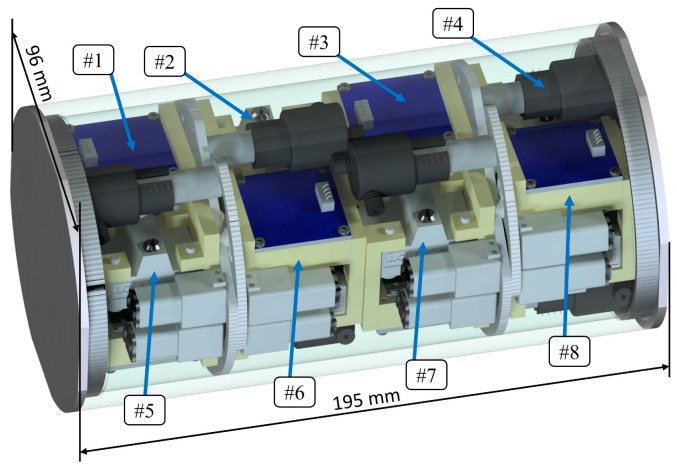
Payload concept containing 8 micropot experiments in a 2U nanosatellite. The numbers indicate the separate micropot experiment modules.

Experiments with the use of simulated microgravity can bring us closer to the behaviour of biological objects in space [13,15,16]. However, on Earth, it is difficult to perform experiments that simulate the simultaneously various factors existing in space, e.g., temperature changes and radiation. Therefore, it is necessary to conduct experiments in real space conditions with minimal overall costs. The CubeSat satellite standard works very well in the implementation of this. Nevertheless, due to the very limited payload volume, the most frequently chosen size of nanosatellites is the 3U standard (over 50% of the launched CubeSat satellites) [19]. In this work, the 2U payload (including CPU) was designed for the two simultaneous growth experiments. However, the system is fully modular and compact, so there is a possibility to fit up to eight experiments at a time in 3U nanosatellites, whereas the payload volume takes 2U and the satellite base takes 1U (Figure 8).

Regardless of the purpose of the research and the chosen standard, it is very important to prepare the samples for departure and for the overloads they are subjected to during the launch of the rocket with the satellite into orbit. The solution presented by us ensured both the safe launch of the experiments into orbit and the utilisation of the most commonly used satellite standard [20,21].

The presented 3D-printed micropot payload construction has the potential and capability to serve as a platform for low orbit microgravity experiments with almost all kinds of seeds. Thanks to the micropot 3D printing fabrication technique, the structure can be freely designed to sustain most of the small seeds. The presented research also confirms that 3D printing can be successfully used as a technique to support some constructional issues in the development of space-oriented devices and instruments [22], but also as a tool for the development of functional devices for astrobiological experiments. Additionally, a critical issue for the further miniaturisation and automation of the bio-payloads is the application of microfluidic circuits (also called as labs-on-a-chip) [23] fabricated by the precise 3D printing techniques involved in the development of microsystems [24].

## 4. Conclusions

The aim of this work was to develop the autonomous system for seed growth experiments under 1 g and simulated microgravity by utilising the 3D-printed micropot and miniaturised instrumentation, fulfilling the requirements of nanosatellite missions. The structure of the payload presented in the article meets all the conditions for seed cultivation. The appropriate culture medium, nutrient supply, temperature, and lighting were provided. In total, 32 seed growth experiments were carried out under simulated microgravity and reference conditions. The influence of the simulated microgravity on seed growth was observed and documented. The works carried out present a ready-to-use device enabling research on the grain cultivation on Earth, using microgravity simulators on Earth and in space. The cress seed cultures presented in the work are an example that illustrate the possibilities of the performed payload.

## Data Availability

Data available under request via e-mail: bartosz.kawa@pwr.edu.pl.
